# Dual role of spreading depolarization in an epileptic focus

**DOI:** 10.1002/epi.70252

**Published:** 2026-04-15

**Authors:** Daria Vinokurova, Karina Tukhvatullina, Roustem Khazipov, Azat Nasretdinov

**Affiliations:** ^1^ Laboratory of Neurobiology Kazan Federal University Kazan Russia; ^2^ Aix‐Marseille University, L’Institut de Neurobiologie de la Méditerranée (INMED), Institut national de la santé et de la recherche médicale (Inserm) Marseille France

**Keywords:** EEG, electrocorticography, epilepsy, postictal depression, seizure initiation, spreading depolarization

## Abstract

**Objective:**

Spreading depolarizations (SDs) are often associated with epileptic discharges. Although SDs are traditionally thought to contribute to postictal depression and termination of epileptic discharges, seizures may also occur during SDs or may even follow SDs, suggesting that interactions between SD and seizures are more complex. Here, we examined the interactions between SD and epileptic activity by spatially separating the epileptic focus and the site of SD initiation.

**Methods:**

Subdural electrocorticographic arrays (6 × 10 electrodes) and intracortical silicon probes were used to record SDs and epileptic activity in the rat parietal cortex. An epileptic focus was induced by local intracortical injection of the potassium channel blocker 4‐aminopyridine combined with the γ‐aminobutyric acid type A receptor antagonist gabazine, whereas extrinsic SDs were evoked by distal high‐potassium solution application.

**Results:**

We found that extrinsic SDs exerted a biphasic effect; they initially promoted seizurelike events (SLEs) when the SD wave approached the epileptic focus, which was then followed by suppression of epileptic activity after the SD spread through the focus. The timing of SLEs relative to SDs varied at different recording sites, with SLEs occurring before, during, or after SD arrival depending on electrode position along the trajectory of SD propagation between the SD initiation site and the epileptic focus. During intracortical recordings, the proconvulsive effects of SD were associated with a wave of pre‐SD neuronal excitation reaching the epileptic focus. The epileptic focus per se also demonstrated resistance to the SD invasion.

**Significance:**

The interactions between SDs and an epileptic focus are not limited to postictal depression, and SDs may also promote epileptic activity in the hyperexcitable cortex.

**Key points**

- Effects of SD on an epileptic focus are dual: both pro‐ and anticonvulsive.
- As extrinsic SDs approach an epileptic focus, they promote epileptic discharges.
- The relative timing of epileptic discharges to SDs varies along the SD trajectory.
- Proconvulsive effects of SDs align with the pre‐SD excitation.
- An epileptic focus resists SD propagation.

## 1 INTRODUCTION

Spreading depolarizations (SDs) are waves of collective depolarization of neurons and glial cells that spread slowly throughout the cerebral cortex.^1^, ^2^, ^3^, ^4^, ^5^, ^6^ Convincing evidence indicates a close association between SDs and epilepsy, both in patients^7^, ^8^, ^9^, ^10^, ^11^, ^12^, ^13^, ^14^, ^15^, ^16^ and in animal models in vivo^8^, ^9^, ^11^, ^17^, ^18^, ^19^, ^20^, ^21^, ^22^, ^23^, ^24^, ^25^ and in vitro^26^, ^27^, ^28^, ^29^, ^30^, ^31^ (for reviews, see Vinogradova,^32^ Aiba,^33^ and Levesque et al.^34^). The prevailing hypothesis is that SDs may be instrumental in postictal depression.^18^, ^19^, ^22^, ^35^, ^36^ In addition, SDs may underlie sudden unexpected death in epilepsy,^24^ headache,^37^, ^38^ the antidepressant effects of electroconvulsive therapy,^8^ and postictal wandering.^9^ However, in addition to the occurrence at the end of epileptic discharges, SDs can also occur during the epileptic discharges,^17^, ^39^, ^40^ as well as before epileptic discharges as in the case of “spreading convulsions,” suggesting that SDs may also promote epileptic activity.^1^, ^7^, ^10^, ^13^, ^29^, ^31^, ^41^, ^42^, ^43^, ^44^, ^45^, ^46^ The wave of pre‐SD excitation is a potential cause of the proconvulsive effects of SD.^47^, ^48^, ^49^ On the other hand, epileptic cortex displays resistance to SD.^50^, ^51^ These observations suggest that the interactions between SD and paroxysmal activity are not limited to postictal depression but are more sophisticated. However, this issue is complicated by the intricate spatiotemporal organization of both epileptic activity and SD initiation and propagation patterns. To circumvent these problems, we used multielectrode electrocorticographic (ECoG) arrays and intracortical silicon probes to examine the interactions between SD and epileptic activity in a model in which SD initiation site and epileptic focus were separated in space.

## 2 MATERIALS AND METHODS

The animal experiments were carried out in compliance with the ARRIVE (Animal Research: Reporting of In Vivo Experiments) guidelines and EU Directive 2010/63/EU for animal experiments, and all animal use protocols were approved by the local ethical committee of Kazan Federal University (#24/22.09.2020). Wistar rats of both sexes aged from 3 to 8 weeks were used. Animals were prepared for head‐restrained recordings under isoflurane anesthesia (4% for induction, 2% for maintenance).^49^ Recordings were performed under urethane anesthesia (1.5 g/kg). A craniotomy of 4 × 5‐mm size was performed over left parietal cortex, and the dura mater was removed. ECoG recordings of the local field potential (LFP) were performed using custom ECoG electrode arrays (6 × 10) in grids of chromium–gold electrodes (50 μm in diameter, spacing = .4 mm^52^). Intracortical LFP and multiple unit activity (MUA) were recorded using two linear multichannel silicon probes with iridium electrodes (413 μm^2^ surface area, 100 μm separation distance; NeuroNexus). The probes were inserted vertically to a depth of 1.6–1.8 mm as follows. The focal probe was placed close to the site of 4‐aminopyridine (4AP)/gabazine injection, whereas the distal one was placed more caudally at a distance of 1.6–2.0 mm from the focal probe. The signals were amplified, lowpass filtered at 9 kHz, and digitized at 32 kHz using a DigitalLynx SX amplifier and Cheetah 6.3.2 (Neuralynx).^53^ A second cranial window ~.3 mm in diameter for SD induction with epidural KCl (.5 mol·L^−1^) application was made over the occipital cortex.^54^ A cocktail of 4‐AP (300 μmol·L^−1^) and gabazine (30 μmol·L^−1^) in a phosphate buffer solution was applied using a Micro4 (WPI) pump through a glass pipette (1 μL/min, total = .5 μL) at a cortical depth of 1000 μm. Intrinsic optical signal (IOS) imaging was performed using an infrared light‐emitting diode installed under the animal's head and a QICAM Fast 1394 CCD camera (QImaging; 1 frame/s, 520 × 696‐pixel resolution).

Raw data were preprocessed using custom‐developed programs in the MATLAB environment. Positive polarity is graphed as up throughout the article. The original direct current (DC) signal was downsampled to 1 kHz for LFP analysis. SD onset was determined from the peak of the first derivative (SD') of the 1 Hz lowpass‐filtered LFP signal during the initial SD depolarization phase.^54^ Events with an SD' peak < 1 mV/s were discarded from the analysis. Spectral power was valued using direct multitaper estimators (1‐Hz bandwidth, 3 tapers, 2‐s spectral window with .1‐s step, Chronux toolbox) for the LFP signal on focal ECoG in the range of 2–70 Hz (alternating current band) during the following intervals: −50 s to 20 s before seizurelike event (SLE) onset, between SLE onset and offset, and 10 s after SLE offset. To detect population spikes (PSs), the first derivative of the average LFP (>.1 Hz) was calculated across all ECoG channels. PSs were then detected as local negative events below a threshold of −100 μV/ms with a minimum next event detection time of 40 ms. The frequency of detected PSs was calculated with a 1‐s sliding window advanced in 1‐ms steps. PS bursts with the PS frequency exceeding a threshold defined by 3 standard deviations for >4 s were considered as an SLE. The SLE onset and offset were defined as time of the PSs closest to the threshold of 1 standard deviation of the PS frequency. For the MUA detection, the original signal was filtered (250–4000 Hz, Daubechies wavelet filter^55^), and negative local peaks >4 standard deviations from the quietest 100‐s fragment in the control period were considered to be MUA.

Statistical analysis was performed using the MATLAB Statistics toolbox. Wilcoxon rank‐sum and signed‐rank two‐sided tests and descriptive statistics^56^ were used to assess the significance of differences between samples. The level of significance was kept at *p* < .05. Pooled data are presented as median, 25th (Q1), and 75th (Q3) percentiles, in the form of boxplots or violin plots. Because SLE occurrence did not depend on SD features (Figure S1), SD order in the cluster (Figure S2), or sex of the animal (Figure S3), and there was no difference in SLE and SD parameters based on sex (Figure S3), these data were pooled together.

## 3 RESULTS

**FIGURE 1:**
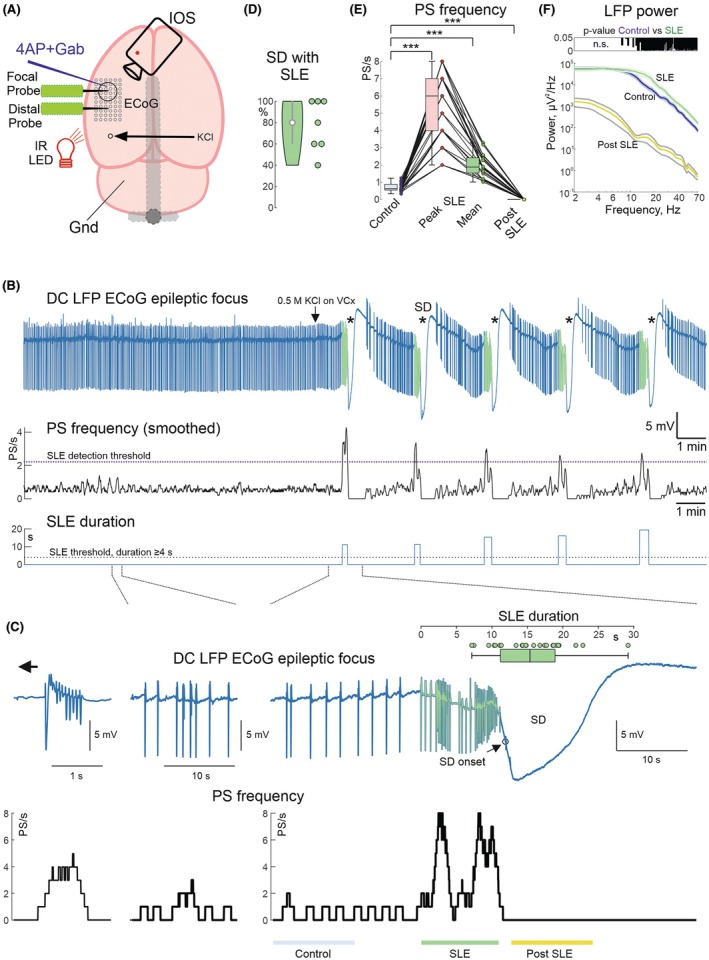
Extrinsic spreading depolarizations (SDs) promote seizurelike activity in the epileptic focus. (A) Scheme of experimental setup for recordings of SDs evoked by distant epipial high‐KCl application on visual cortex (VCx) and activity in the epileptic focus induced by local intracortical injection of a cocktail of 4‐aminopyridine (4AP) and gabazine (Gab). The gray bar represents the metal head‐fixation holder. (B) Cluster of five recurrent SDs (marked by asterisks) recorded from an epileptic focus. From top to bottom: direct current (DC)–electrocorticographic (ECoG) recordings from an electrode adjacent to the injection site (blue trace) and population spike (PS) frequency plot; seizurelike events (SLEs) are colored green. (C) Left: Two examples of PS bursts shown on an expanded time scale. These bursts were subthreshold for the SLE detector. A left cloniclike event occurred prior to the fragment shown in panel B (note that time scale is different from the right traces). Right: The first SD episode and SLE in the SD cluster induced by distant KCl application. Top horizontal boxplot indicates SLE duration (median with 25th and 75th percentiles) with time = 0 corresponding to the SLE onset. Each dot corresponds to an individual SD. (D) Probability of SLE occurrence in association with SD. Each dot corresponds to an individual animal. (E) PS frequency before SD (control, blue), the peak value during SD‐associated SLE (pink), the mean value during SLE (green), and after SLE (yellow). (F) Local field potential (LFP) power spectral density (on a logarithmic scale) in the 2–70‐Hz range, calculated for the ECoG channel above the epileptic focus before SD (control, blue), during SLE (green), and after SLE (yellow). Top: Probability value plot for a difference between the LFP before and during SLE (two‐way Wilcoxon signed rank test). C–F: Pooled data from *n* = 24 SDs recorded from seven rats. Gnd, ground; IOS, intrinsic optical signal; IR, infrared; LED, light‐emitting diode. *** *p* = 1.8×10^−5^

We explored the effects of SD on epileptic activity in cerebral cortex by separating in space the epileptic focus and the site of SD induction (Figure 1A). An epileptic focus was induced by an intracortical injection of the potassium channel blocker 4AP and γ‐aminobutyric acid type A receptor blocker gabazine. Epileptic activity in the focus was characterized by regular interictal PSs occurring at a frequency of median = .77 (Q1–Q3 = .61–1.00) Hz and attaining median = 9.8 (Q1–Q3 = 6.1–10.0) mV (*n* = 7 rats; Figure 1B,C, Video S1). SDs were evoked by focal epidural application of high‐potassium (.5–1 mol·L^−1^) solution on visual cortex at 3–4 mm caudal from the epileptic focus. This induced recurrent SDs associated with characteristic negative DC shifts (Figure 1B,C). Remarkably, the vast majority of SDs (~80%; Figure 1D) were also associated with SLEs manifested by bursts of PSs with median = 15.4 (Q1–Q3 = 11.2–18.9) s duration (Figure 1C, top panel), in which PS frequency rose to the peak value of median = 6 (Q1–Q3 = 4–7) PSs/s (mean intraburst PS frequency = 1.9 [Q1–Q3 = 1.5–2.4] PS/s; Figure 1E). Of note, in the present model, the tonic–clonic phenotype was not observed either before or during SD induction, whereas PS bursts similar to SD‐associated SLEs were only barely observed before SD induction (Figure 1С). In some cases, SLEs were associated with two peaks in the increase in PS frequency (Figure 1B,C). SLEs were also associated with an increase in LFP power in the frequency range from 12 to 70 Hz (Figure 1F). All SLEs were characterized by progressive waning of PS amplitude at the end of the SLE, and they were invariably followed by complete suppression of activity in the epileptic focus, characteristic of spreading depression causing postictal depression (Figure 1B,C,E,F).

**FIGURE 2:**
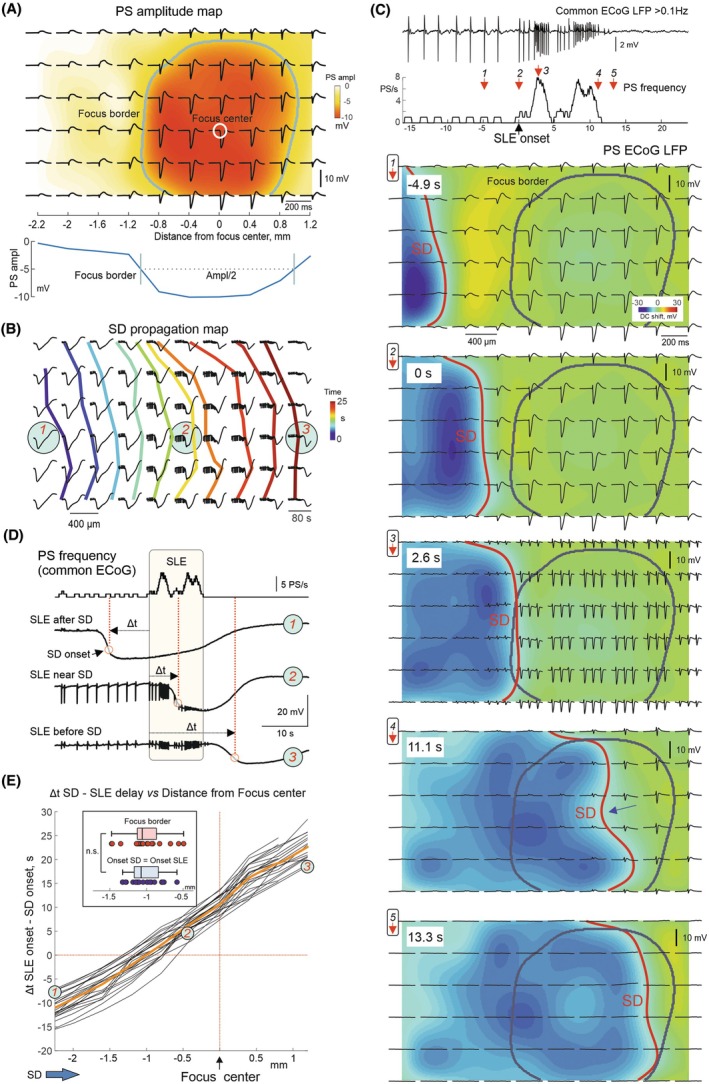
Spreading depolarization (SD) triggers a seizurelike event (SLE) upon reaching the epileptic focus. (A) Average interictal population spike (PS) on 60 electrocorticographic (ECoG) channels (black) overlaid on the color‐coded PS amplitude (ampl) map. The center of the epileptic focus (white circle) is assigned to a channel with the maximal PS amplitude. The epileptic focus border (concentric gray line) delineates the region with greater than half‐maximal PS amplitude as shown on the plot below. (B) Direct current (DC)–ECoG local field potentials (LFPs) during SD induced by high KCl application 5 mm left of the array and propagating through the epileptic focus. Isochrones of SD onsets are indicated by color‐coded lines. (C) Top, common ECoG LFP and corresponding PS frequency plot during a high KCl‐induced SLE. Below, examples of PSs (black) overlaid on color‐coded DC‐ECoG LFPs, and SD onset isochrones (red lines) at five time points indicated by red arrows on the PS frequency plot above. The focus border is indicated by the gray line. The time after distant KCl application is shown in the top left insets. Example #4: The blue arrow shows the slowing of SD in the focus. (D) PS frequency plot with DC‐LFPs recorded from three selected ECoG electrodes on an SD trajectory marked by cyan circles in panel B. Note that SD onsets (vertical dashed lines) occur before SLE (yellow rectangle) on electrode 1, during SLE on the electrode 2, and after SLE on the electrode 3. (E) Relationship of the time lag of SD onset from the SLE onset (Δ*t*) with the distance from the center of the epileptic focus in an ECoG row closest to the focus center. Each black line shows an individual SD; the orange line shows the average. Highlighted circles 1, 2, and 3 correspond to the examples presented in panels B and D. The blue and red boxplots show the distance from the epileptic focus center where the SD and SLE onsets coincide (Δ*t* = 0) and the distance from the focus borders to the focus center, respectively (*p* = .375, two‐way Wilcoxon signed rank test); outliers are not shown. (A–D) Recordings from one rat. (E) Pooled data from *n* = 23 SDs recorded from seven rats.

Although the findings above indicate that extrinsic SDs promote SLEs in the epileptic focus, they also raise a question of where the “hot‐spot” of an SD‐triggered SLE is located. Using ECoG arrays, we first determined the spatial features of the epileptic focus. PS amplitude was maximal on approximately five channels around the site of the injection of blockers (the focus center) and decreased at more remote channels (Figure 2A). The epileptic focus border was defined as half‐maximal PS amplitude, revealing a round epileptic focus of median = .94 (Q1–Q3 = .74–1.09) mm diameter. Importantly, PSs were highly synchronized on all electrodes of the ECoG array (Figure 2A). Next, we assessed SD propagation by monitoring the onset of SD‐related DC shifts on ECoG electrodes and concomitant IOS recordings (Figure 2B, Video S2). On average, SDs propagated at a speed of 5.9 (Q1–Q3 = 5.4–6.3) mm/min but slowed down in the epileptic focus (see below). Snapshots illustrating epileptic activity on the ECoG array at different times of SD propagation are shown in Figure 2C (SLE onset = 0). Interictal activity remained unchanged while SD propagated through the cortex distant from the epileptic focus (Figure 2C, *T* = −4.9 s), and SD in these recording sites largely preceded SLE (Figure 2B,D, ECoG #1; Video S3). However, as soon as SD attained the border of the epileptic focus, it triggered an SLE (Figure 2C, *T* = 0 s and 2.6 s), with SD at these electrodes occurring close to SLE onset (Figure 2B,D, ECoG #2). Further SD propagation through the epileptic focus was associated with the suppression of SLEs (Figure 2C, *T* = 11.1 s and 13.3 s), and SD at these electrodes occurred after SLEs (Figure 2B,D, ECoG #3). We further quantified the delay between the SLE and SD onsets (ΔtSD‐SLE) as a function of the distance from the center of the epileptic focus along the SD trajectory toward the center of the epileptic focus (row 4 in Figure 2B,C,E). At the left electrodes (near the SD initiation site and distant from the epileptic focus), SLEs were strongly delayed after SD. At electrodes near the border of the epileptic focus, SLEs occurred with minimal delay from SD. At the center of the epileptic focus and at remote electrodes along the SD propagation path, SLEs largely preceded SDs (Figure 2E). On average, SLE onset was observed at the time when SD attained a distance of −1.07 (Q1–Q3 = −1.17 to .84) mm from the center of the epileptic focus, matching the location of the epileptic focus border (−1.06 [Q1–Q3 = −1.12 to .87] mm) on the SD‐entry side (Figure 2E, top boxplots). The conclusion from these findings is twofold: (1) the timing of SLE onset from SD largely varies among cortical sites depending on the distance from the epileptic focus and (2) an SLE is initiated when an SD approaches the epileptic focus and reaches its border. Epileptic activity was suppressed in the regions invaded by SD both in the epileptic focus and beyond, and a few minutes after recovery from SD, the interictal activity returned to pre‐SD levels. Of note, in addition to SDs originating from the potassium application site, occasional SDs originating elsewhere, including the epileptic focus, were also associated with SLEs (Video S4, Figure S4).

**FIGURE 3:**
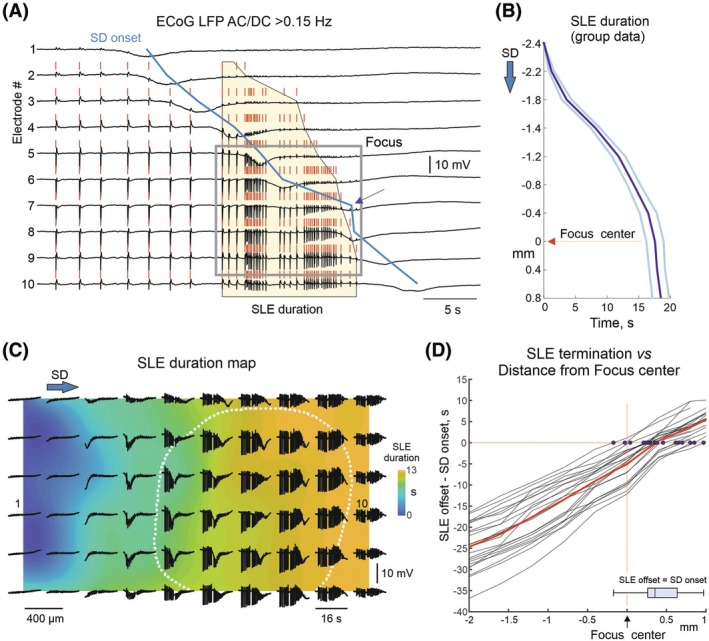
Spreading depolarization (SD) terminates a seizurelike event (SLE) and determines SLE duration along the SD trajectory. (A) Example recordings of alternating current (AC)–electrocorticographic (ECoG) local field potential (LFP; black) in a selected electrode row on an SD trajectory from SD initiation site to the center of the epileptic focus, showing population spikes (vertical red tics), SD onsets (blue line), and focus border (gray box). Note that the SLE (yellow area) terminates earlier and the SLE has a shorter duration at the electrodes closer to the SD initiation point (top channels). The blue arrow shows the slowing of SD in the focus. (B) Dependence of SLE duration on the distance from the focus center along the trajectory of SD propagation (mean ± standard error). (C) Examples of AC‐ECoG LFP during an SD‐induced SLE overlaid on the color‐coded SLE duration map. The border of the epileptic focus is outlined by the dashed gray line. (D) The dependence of the time lag of SLE termination from the SD onset on the distance from the epileptic focus center. The blue boxplot shows the distance from the epileptic focus center where the SD onset coincides with the SLE termination, that is, the SD coordinate where SLE terminated. (A, C) Recordings from one rat. (B, D) Pooled data from *n* = 24 SDs recorded from seven rats. DC, direct current.

Whereas SD‐induced SLE onset was simultaneous on the entire ECoG array, the termination of SLE and SLE duration varied between the electrodes. As shown on example recordings from a row of electrodes indicating SD trajectory from the SD initiation site to the epileptic focus (Figure 3A), SLEs terminated much earlier and lasted for a shorter time on the electrodes located closer to the SD initiation site, whereas at the electrodes located closer to and within the epileptic focus, SLEs terminated later and lasted for a longer time (Figure 3A,B). Of note, short‐duration SLEs at the electrodes located outside the epileptic focus proximal to the SD initiation site (ECoGs #2–#4) occurred after the SD entry to these locations, reminiscent of the “spreading convulsions” phenotype.^10^ Similarly, SLE also persisted after SD onset for up to 10 s at sites located at the border and within the epileptic focus proximal to the SD entry (ECoGs #5–#6). Post‐SD SLEs at these locations were likely supported by the activity in deep layers, which are invaded by SD at a delay from the surface (see below). Increase in SLE duration along the SD trajectory was evident on the entire ECoG array (Figure 3C). Total SLE termination occurred when SD invaded most of the epileptic focus and attained median = .35 (Q1–Q3 = .26–.64) mm from the epileptic focus center toward the SD exit (Figure 3D, Figure 2C, *T* = 11.1 s and 13.3 s). This happened even though the epileptic focus showed relative resistance to SD propagation (see below). Thus, SLE duration strongly varies along the SD trajectory, and SLEs fully terminate when SD invades most of the territory of the epileptic focus, suppressing the critical mass of the epileptic network required for an SLE.

**FIGURE 4:**
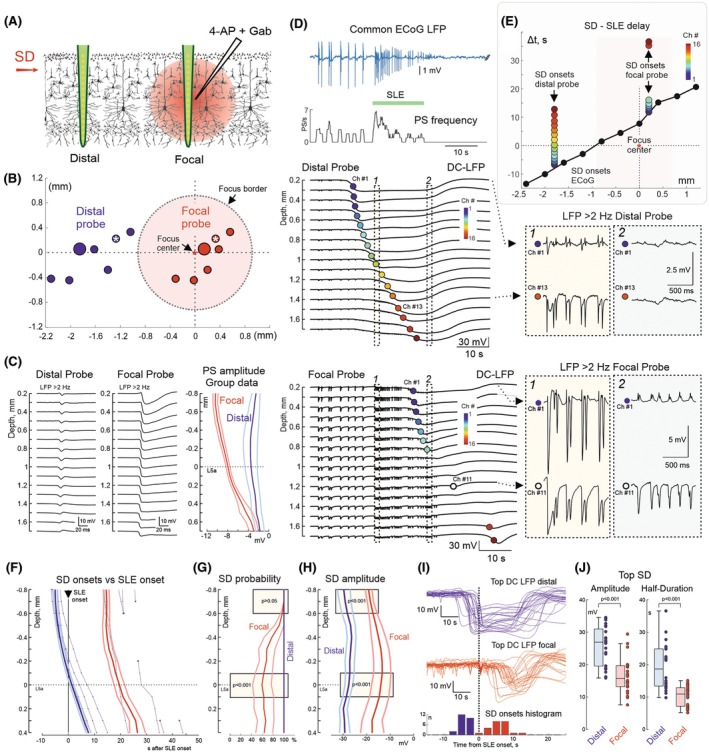
Delay of a seizurelike event (SLE) from spreading depolarization (SD) depends on cortical depth. (A) Sideview scheme of dual direct current (DC)–local field potential (LFP) recordings across cortical depth using one silicon probe inserted distally from the epileptic focus closer to the SD induction site and the second probe inserted into the epileptic focus. (B) View from above of the distal (blue) and focal (red) probes' location relative to the epileptic focus center for all experiments. Shaded circle represents the grand average epileptic focus border (*n* = 7 animals). (C) Depth profile of a population spike (PS) at distal (left) and focal (right) 16‐channel probes. On the right, PS amplitude is shown at different cortical depths (mean ± standard error) at distal (blue) and focal (red) probes. (D) Left: Common alternating current (AC)–electrocorticographic (ECoG) LFP and PS frequency plot, and DC‐LFP recordings through the cortical depth on the distal (middle) and focal (bottom) probes. Colored circles indicate SD onsets, color codes the channel number. Right: Examples of AC‐LFP recordings from channels #1 and #13 of the distal probe and channels #1 and #11 of the focal probe during the two highlighted time segments (episodes 1 and 2) on expanded time scale. (E) Dependence of the time lag of SD onset from the SLE onset on the distance from the center of the epileptic focus in an ECoG row closest to the focus center (black) and at the color‐coded cortical depth of the distal and focal probes shown in panel D. (F) Depth profile of SD onset at distal (blue) and focal (red) probes. Four examples are shown for one animal. Group data are presented relative to the channel in layer L5. Circles indicate the SDs interrupted in the middle of the cortex. (G) Depth profile of SD occurrence across cortical depth. (H) Depth profile of the SD amplitude in focal (red) and distal (blue) probes (mean ± standard error). (I) Superimposed DC‐LFPs from the top channel of the distal (blue) and focal (red) silicon probes and corresponding histogram of SD onsets relative to SLE onset. (J) Amplitude and duration of SDs at the top channel of the distal and focal probes (*p* = 7.5e‐4 and *p* = 4.9e‐5, respectively; two‐way Wilcoxon signed rank test). (A) The cortex texture is adapted from Wells.^61^ (E–J) Pooled data from *n* = 24 SDs recorded from seven rats. 4‐AP, 4‐aminopyridine; Gab, gabazine.

Because SD spreads not only in horizontal but also in vertical cortical space,^17^, ^49^, ^54^, ^57^, ^58^, ^59^, ^60^ we further explored the relationships between SD and SLEs by recording activity through the cortical depth using silicon probes. One of them was inserted into the “naïve” cortex on the path of the SD toward the epileptic focus (“distal” probe), and another probe was inserted into the epileptic focus (“focal” probe; Figure 4A,B). The amplitude of interictal PSs was maximal at the focal probe, with the largest PS amplitude observed in the superficial layers (Figure 4C). The DC shifts during SDs on the top channels of the intracortical probes were similar to the DC shifts of SDs recorded from the nearby ECoG electrodes (Figure 4D,E). In keeping with the top‐down gradient of vertical SD propagation,^17^, ^49^, ^54^, ^59^, ^60^ SDs spread from the superficial to deep layers on both probes (Figure 4D). As a result of the vertical SD propagation, the timing of SLEs relative to SDs also varied depending on cortical depth. As shown in the example recordings at the distal probe (Figure 4D), SD started earlier than SLEs on the top channels, coincided with SLE onset at the middle channels, and arrived approximately 10 s after SLE onset in the deepest channels (Figure 4D,E). At the probe in the epileptic focus, SD started after SLE onset at all cortical depths, but the SD–SLE delay also strongly increased with cortical depth (Figure 4D,E). The diversity in SD delays from SLE onset and their increase with cortical depth were evident at the group data level (Figure 4F).

**FIGURE 5:**
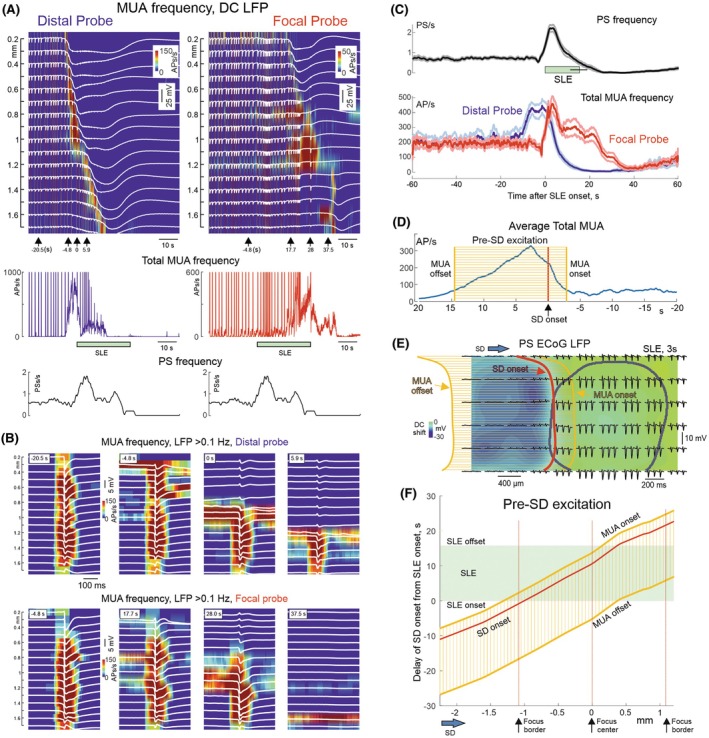
Wave of pre‐spreading depolarization (SD) excitation and seizurelike events (SLEs). (A) Color‐coded multiple unit activity (MUA) density map across cortical depth during SD recorded by distal (left) and focal (right) silicon probes. White traces indicate direct current (DC)–local field potential (LFP) at different cortical depth. Silicon probe positions are indicated by white asterisks in Figure 4B. Bottom: Corresponding total MUA frequency across all depth and common electrocorticographic (ECoG) population spike (PS) frequency plots. (B) Example PSs at different time points from SLE onset (as indicated by bottom arrows in panel A) along SD propagation through the cortical column recorded by distal (top) and focal (bottom) probes. (C) Grand average of common ECoG PS frequency (top) and total MUA frequency (bottom) at distal (blue) and focal (red) probes aligned to the SLE onset. (D) Grand average of total MUA frequency aligned to the surface SD onset. Boxplot shows the pre‐SD total MUA burst onset and offset times. (E) Reconstruction of the pre‐SD excitation (yellow grid) at the 3rd second of SLE in the horizontal cortical plane. Template of pre‐SD excitation (panel D) was triggered by surface SD onset during ECoG recordings (SD onset, red line). DC‐ECoG LFP is coded by background color (blue, SD‐related negative DC shift). Black traces indicate 200‐ms fragments of alternating current (AC)–ECoG LFP. Gray line indicates epileptic focus border. (F) Propagation of the reconstructed pre‐SD excitation wave in coordinates of SD–SLE onset delay (y‐axis; gray area shows the entire SLE period) along the SD trajectory from the SD initiation site through the center of the epileptic focus (x‐axis; focus center and borders are indicated by bottom arrows and vertical red lines). (C–F) Pooled data from *n* = 24 SDs recorded from seven rats.

During their vertical propagation, SDs created transient states with unique organization of epileptic activity within a cortical column. Two examples of such transient states, during which SD attained the middle depth at the distal probe (Example #1) and the focal probe (Example #2), respectively, are shown in Figure 4D (right panels). In Example #1, PSs were suppressed or even changed the polarity from negative to positive in the superficial layers already invaded by SD (Ch #1) while maintaining negativity in deeper layers not yet invaded by SD (Ch #13) on the distal probe, as well as at all depths of the focal probe (for comparison with the pre‐SD state, see Figure 4C). In Example #2, epileptic activity was blocked by SD across all channels of the distal probe, whereas on the focal probe, PSs switched the polarity from negative to positive in the superficial layers invaded by SD (Ch #1) while maintaining negativity in deeper layers (Ch #11). The changes in the electrographic phenotype of the PSs during vertical SD propagation were also associated with a suppression of neuronal activity (MUA) in the superficial but not deep layers (Figure 5A,B).

Interestingly, whereas full SDs on the distal probe displayed a vertical propagation profile with SDs sequentially invading all layers similar to that in normal cortex, SDs in the epileptic focus showed a tendency to avoid middle cortical depth with eventual interruption of SDs in the middle layers near the depth of the proconvulsant injection (Figure 4D,F). During group analysis, vertical position of the silicon probe channels was normalized to the depth of layer 5, the hallmark of which is the highest neuronal firing level.^62^, ^63^, ^64^, ^65^ The tendency of SD to avoid middle layers of the focus manifested in lower SD probability in the middle and deep layers compared to the superficial depth in the focus and to the distal probe (Figure 4F,G) and the smallest SD amplitude at the middle depth of the focus (Figure 4H). The similar tendency of SD to avoid the epileptic focus was also supported by smaller SD amplitude through the entire depth of the focus compared to distal sites (Figure 4H) and smaller SD amplitude and shorter SD duration at the top channels of the silicon probes in the epileptic focus (Figure 4I,J). Moreover, a slowing of SD propagation at the focus was also observed during propagation in the horizontal plane (Figure 2C, Example #4; Figure 3A, arrows). Although a certain degree of damage to the cortex by injection of blockers cannot be excluded, the depth profile of PSs in the focal probe (Figure 4C) as well as persistence of MUA in middle cortical layers in the core of the focus (Figure 5A,B) favor the idea that epileptic cortex shows resistance to SD that is in agreement with findings in other epilepsy models and in the human epileptic cortex.^50^, ^51^

The present findings indicate that the initiation of SLEs is associated with SD entering the epileptic focus. What are the mechanisms underlying seizures triggered by SD in the epileptic focus? Although SD causes nearly complete loss of membrane potential, depolarization block, and full suppression of all forms of activity, these phenomena do not occur in an all‐or‐none manner but gradually. SD is preceded by a pre‐SD excitation phase, characterized by moderate, near‐threshold neuronal depolarization and increased neuronal activity,^19^, ^47^, ^49^, ^54^, ^57^, ^66^ so pre‐SD excitation could potentially be involved in the proconvulsive effect of SD in the epileptic focus. Therefore, we further explored spatial–temporal organization of neuronal activity during spread of SD through the epileptic focus using silicon probe MUA recordings (Figure 5). We found that SDs are preceded by a wave of pre‐SD excitation first on the distal and then on focal silicon probes, characterizing a top‐down temporal organization corresponding to the vertical SD propagation (Figure 5A,B). Although neuronal firing during the pre‐SD period was almost completely synchronized in PSs, pre‐SD excitation was also associated with elevation of MUA in the inter‐PS intervals, along with MUA suppression during both PSs and inter‐PS intervals in the layers invaded by SD (Figure 5B). Although the pre‐SD excitation, assessed as total MUA across all layers of the cortical column, largely overlapped in time with SLEs, it was delayed in the focal probe from the distal probe along with a horizontal SD propagation (Figure 5C). We further attempted to characterize the pre‐SD excitation dynamics during SD propagation through the epileptic focus in the horizontal cortical plane. In this aim, we built a template of pre‐SD excitation based on total MUA assessment using the silicon probe recordings merging data from the distal and focal probes relative to SD onset detected on the cortical surface (Figure 5D). In the horizontal plane, although pre‐SD excitation within a column (total duration 17.4 s) started shortly before the surface SD (−2.9 s), the peak and vast majority of pre‐SD excitation occurred after the SD onset at the cortical surface (+14.5 s; Figure 5D). We next plotted the template of pre‐SD excitation on the map of horizontal SD propagation taking SD onsets detected on ECoG electrodes as local time reference points (Figure 5E, Video S5). This enabled us to reconstruct propagation of the pre‐SD excitation wave in the horizontal cortical plane (a reconstruction at the third second of an SLE is shown in Figure 5E) and to relate pre‐SD excitation to the coordinates of the epileptic focus in horizontal cortical space, as well as to the SLE onset and offset in time. Results of such analysis in a reduced dimension of SD trajectory from the SD initiation site through the center of the epileptic focus are shown on Figure 5F. It is noticeable that the SLE onset is associated with entry of pre‐SD excitation wave into the epileptic focus, and that propagation of the pre‐SD excitation through the epileptic focus occurs through the entire time course of the SLE. It may seem paradoxical that pre‐SD excitation within the focus culminates at the end of the SLE. However, the pre‐SD excitation wave is paralleled by a wave of SD‐related suppression of activity, which propagates from superficial to deep layers (Figure 5B,C), reducing the mass of the epileptic network. Therefore, it is plausible that SLE termination is caused by SD‐induced depression of activity within a critical mass of the epileptic network despite persisting pre‐SD excitation in the deep layers of cortex non yet invaded by SD.

## 4 DISCUSSION

Our main finding is that SD exerts a dual and biphasic role in the epileptic focus; although extrinsic SDs initially promote epileptiform activity when the SD approaches the epileptic focus, they also terminate seizures when SD fully invades the epileptic focus. These findings also provide a mechanistic explanation for the diversity in temporal relationships between SD and SLEs, with SLEs following SD, occurring during SD and preceding SD at different cortical sites relatively the trajectory of SD propagation.

The present findings suggest the following model of the complex and dynamic interactions between SD and epileptic focus. Activity in the hyperexcitable network of epileptic focus is organized in regular interictal PSs at the resting state prior to SD. A wave of SD, generated far away from the focus, slowly propagates toward the epileptic focus, causing a sequence of pre‐SD excitation followed by depression in the cortical territories recruited by SD.^47^, ^48^, ^49^ Pre‐SD excitation does not cause epileptic activity in the “naïve” cortex, where inhibition efficiently balances excitation.^27^, ^49^, ^66^ Of note, in the hippocampus the prodromal pre‐SD excitation may be organized in brief and local large‐amplitude PS bursts.^48^, ^67^, ^68^ At this stage, activity in the epileptic focus remains unchanged, and SD in these regions distant from the focus precede a forthcoming SLE. However, when SD approaches the focus border, pre‐SD excitation causes an SLE in the epileptic focus where inhibition is suppressed.^69^, ^70^, ^71^, ^72^ The mechanism of proconvulsive effect of SD is likely to be fundamentally similar to that of pre‐SD excitation, and involves mild neuronal depolarization at the SD onset caused by elevation of extracellular potassium, increase in neuronal firing, and activation of local excitatory connections and nonsynaptic glutamate release, as well as ephaptic interactions.^2^, ^3^, ^4^, ^73^, ^74^

An SLE lasts as long as pre‐SD excitation propagates through the epileptic focus, followed by a wave of spreading depression resulting in SLE termination, when SD invades most of the epileptic focus, suggesting that a critical mass of neuronal network in the focus is required for SLE generation. Whereas SLE onset is highly synchronous in the entire epileptic focus, SLE termination is a dynamic process determined by SD propagation in both horizontal and vertical cortical space. Horizontal SD propagation through the epileptic focus creates transient states during which epileptic activity is suppressed across the entire cortical depth whereas an SLE persists in the regions of the epileptic focus in front of SD propagation. As a result, SLE duration differs within the focus, being shortest in the regions first invaded by SD and longest in more distant regions not yet invaded by SD along the SD trajectory. In keeping with the top‐down gradient in SD propagation across cortical depth, SD first recruits superficial layers, which also creates transient network states in the vertical dimension. Although PSs normally involve the entire cortical column, SD first suppresses PSs in the superficial layers, but epileptic activity persists in deep layers. Change in PSs from negative to positive polarity is a hallmark of this transient state with the partial SD invasion to the superficial layers.^17^ This phenomenon likely involves a suppression of the superficial generator, which predominantly contributes to PS negativity at the cortical surface, and unmasking of the positive passive source of the deep PS generator, which is mechanistically similar to a boost in surface delta power during partial superficial SDs.^49^

We found that the core of the epileptic focus is resistant to SD propagation. This is in keeping with previous findings in other models, in which SDs similarly avoided penetration to the epileptic focus.^51^ Similarly, slices of chronically epileptic human and rat neocortex display resistance against SD in vitro.^50^ Interestingly, resistance to SD was manifested not only by a reduced SD amplitude and duration, as well as by slowing of horizontal SD propagation in the epileptic focus, but also by low penetration rate and smaller SD amplitude in the core of the epileptic focus, at the depth of intracortical injection of the proconvulsant cocktail. Although the mechanisms of higher resistance of the epileptic cortex to SD remain to be elucidated, resistance of the epileptic focus to SD should be considered during SD detection in the epileptic patients.

## 5 CONCLUSIONS

Our results suggest complex and dynamic interactions between SDs and the epileptic focus, in which SDs exert dual pro‐ and anticonvulsive roles. Although SDs clearly contribute to the postictal depression and termination of epileptic discharges, they may also promote seizures in the epileptic focus through the synergistic interaction of pre‐SD excitation with hyperexcitable networks of the epileptic focus. Our results also point to the highly dynamic nature of the interactions between SD and epileptic focus, in both horizontal and vertical cortical space, which creates unique transient network states with distinct electrophysiological phenotypes. Present findings could be useful in clinical investigations and interpretation of the electrophysiological data in epileptic patients and in other pathological states associating SD and epileptiform activities.

## AUTHOR CONTRIBUTIONS

*Conceptualization:* Roustem Khazipov and Azat Nasretdinov. *Methodology:* Azat Nasretdinov, Daria Vinokurova, and Roustem Khazipov. *Investigation:* Daria Vinokurova, Karina Tukhvatullina, and Azat Nasretdinov. *Visualization:* Azat Nasretdinov, Daria Vinokurova, Karina Tukhvatullina, and Roustem Khazipov. *Funding acquisition:* Roustem Khazipov and Azat Nasretdinov. *Project administration:* Roustem Khazipov and Azat Nasretdinov. *Supervision:* Roustem Khazipov, Azat Nasretdinov, and Daria Vinokurova. *Writing—original draft:* Roustem Khazipov and Azat Nasretdinov. *Writing—review and editing:* Daria Vinokurova, Karina Tukhvatullina, Roustem Khazipov, and Azat Nasretdinov.

## FUNDING INFORMATION

The research was supported by RSF grant 22‐15‐00236‐P.

## CONFLICT OF INTEREST STATEMENT

The authors declare no competing interests. We confirm that we have read the Journal's position on issues involved in ethical publication and affirm that this report is consistent with those guidelines.

## Supporting information


Figure S1.

Figure S2.

Figure S3.

Figure S4.

Video S1.

Video S2.

Video S3.

Video S4.

Video S5.

## Data Availability

Animal data supporting the results of the study are available from the authors upon request. An earlier version of this article is available on *bioRxiv*.^75^ Codes for the data analysis are available from the authors upon request.
