# Supplementary material for: Dual role of spreading depolarization in an epileptic focus
Source: Epilepsia. 2026 Apr 15;67(7):3815–28. doi: 10.1002/epi.70252 (PMC13360997; doi:10.1002/epi.70252)
Supplement: Supplementary file 1 — Figure S1. [file EPI-67-3815-s004.docx]

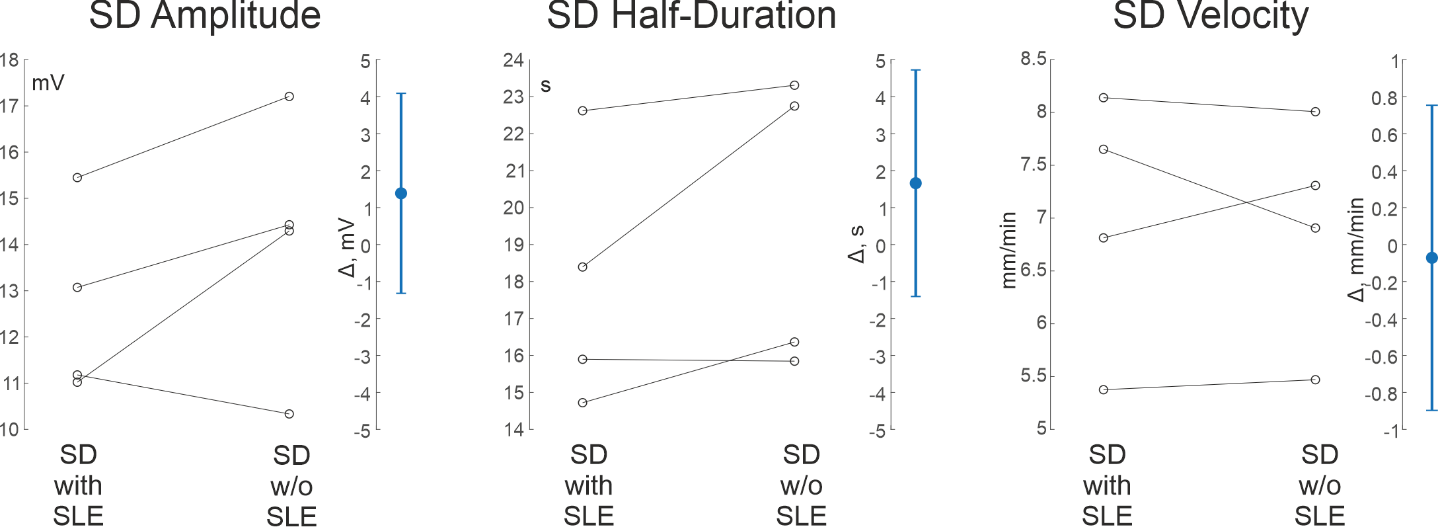


**Supplementary Figure S1. Estimation statistics for SD parameters for the SD cases with and without SLE.**

The plots show the SD parameters (SD amplitude, SD half-duration, and SD velocity) on the ECoG channel near the epileptic focus for the SD cases with and without SLE. The data presented herein pertain to four animals in which the probability of SLE during SD was less than 100%. Each circle represents the mean value within an animal. To the right of each panel, the mean difference with confidence intervals is shown, indicating the effect size.
