# Supplementary material for: Dual role of spreading depolarization in an epileptic focus
Source: Epilepsia. 2026 Apr 15;67(7):3815–28. doi: 10.1002/epi.70252 (PMC13360997; doi:10.1002/epi.70252)
Supplement: Supplementary file 2 — Figure S2. [file EPI-67-3815-s008.docx]

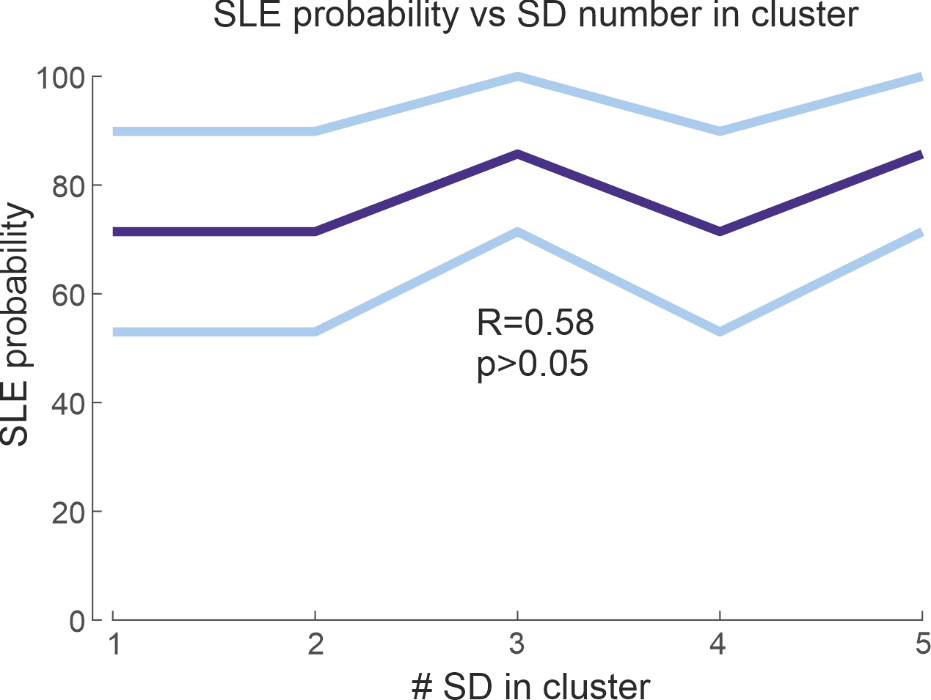


**Supplementary Figure S2. The probability of SLE does not depend on the SD number in a cluster.**

The graph shows the probability of SLE (mean ± standard error) as a function of SD number in a cluster. Group data for n=7 animals.
