# Supplementary material for: Dual role of spreading depolarization in an epileptic focus
Source: Epilepsia. 2026 Apr 15;67(7):3815–28. doi: 10.1002/epi.70252 (PMC13360997; doi:10.1002/epi.70252)
Supplement: Supplementary file 3 — Figure S3. [file EPI-67-3815-s002.docx]

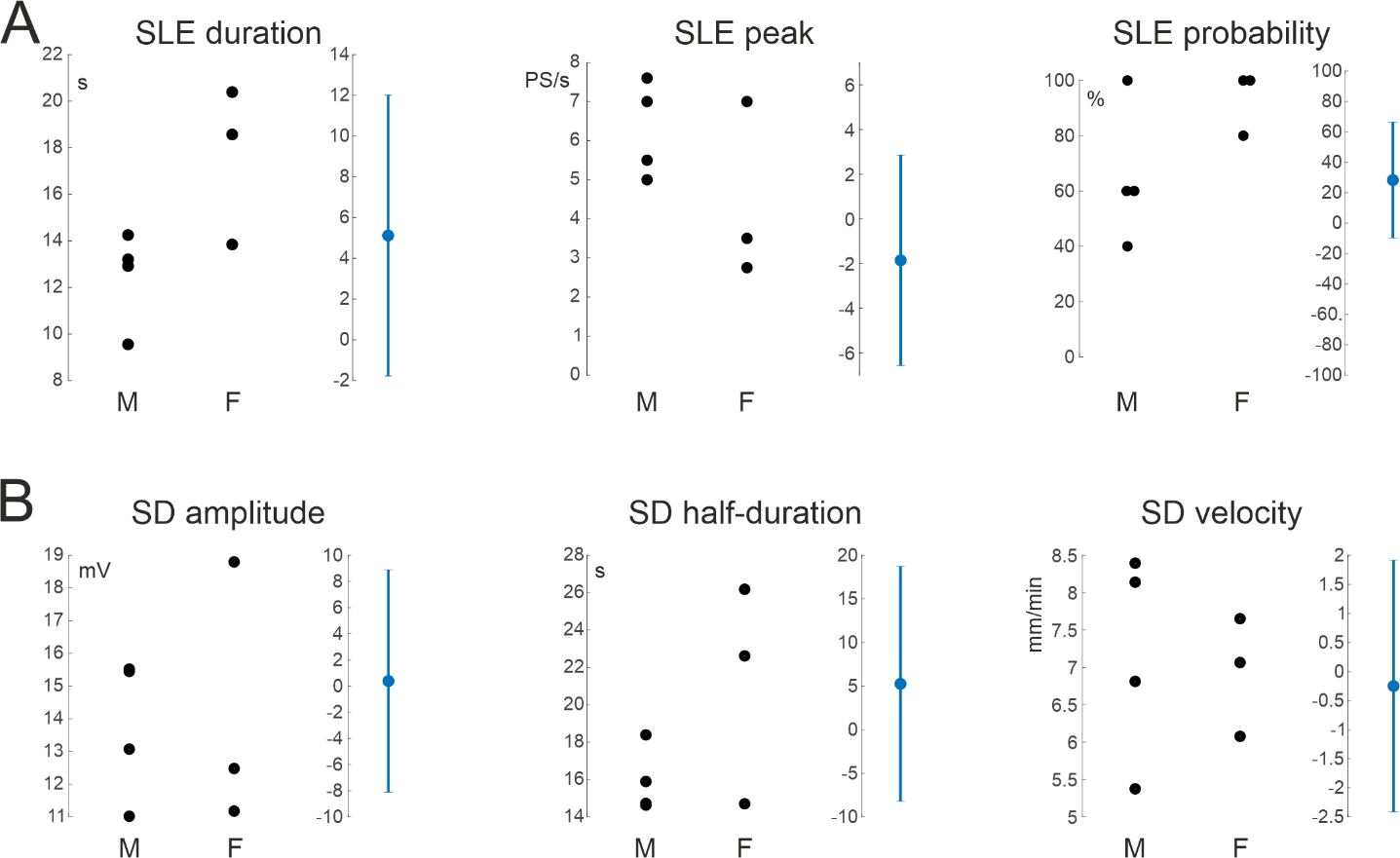


**Supplementary Figure S3. The SD and SLE parameters don’t depend on the animal’s sex.**

(**A**) SLE parameters (duration, peak value, probability) in male (M) and female (F) rats. Values were averaged for each animal. To the right of each panel, the mean difference and confidence intervals are shown, indicating the effect size. (**B**) SD parameters (amplitude, half-duration, velocity) on the ECoG channel near the epileptic focus in male and female rats. To the right of each panel, the mean difference and confidence intervals. A-B: Group data for n=7 animals (four males and three females).
