# Supplementary material for: Dual role of spreading depolarization in an epileptic focus
Source: Epilepsia. 2026 Apr 15;67(7):3815–28. doi: 10.1002/epi.70252 (PMC13360997; doi:10.1002/epi.70252)
Supplement: Supplementary file 4 — Figure S4. [file EPI-67-3815-s006.docx]

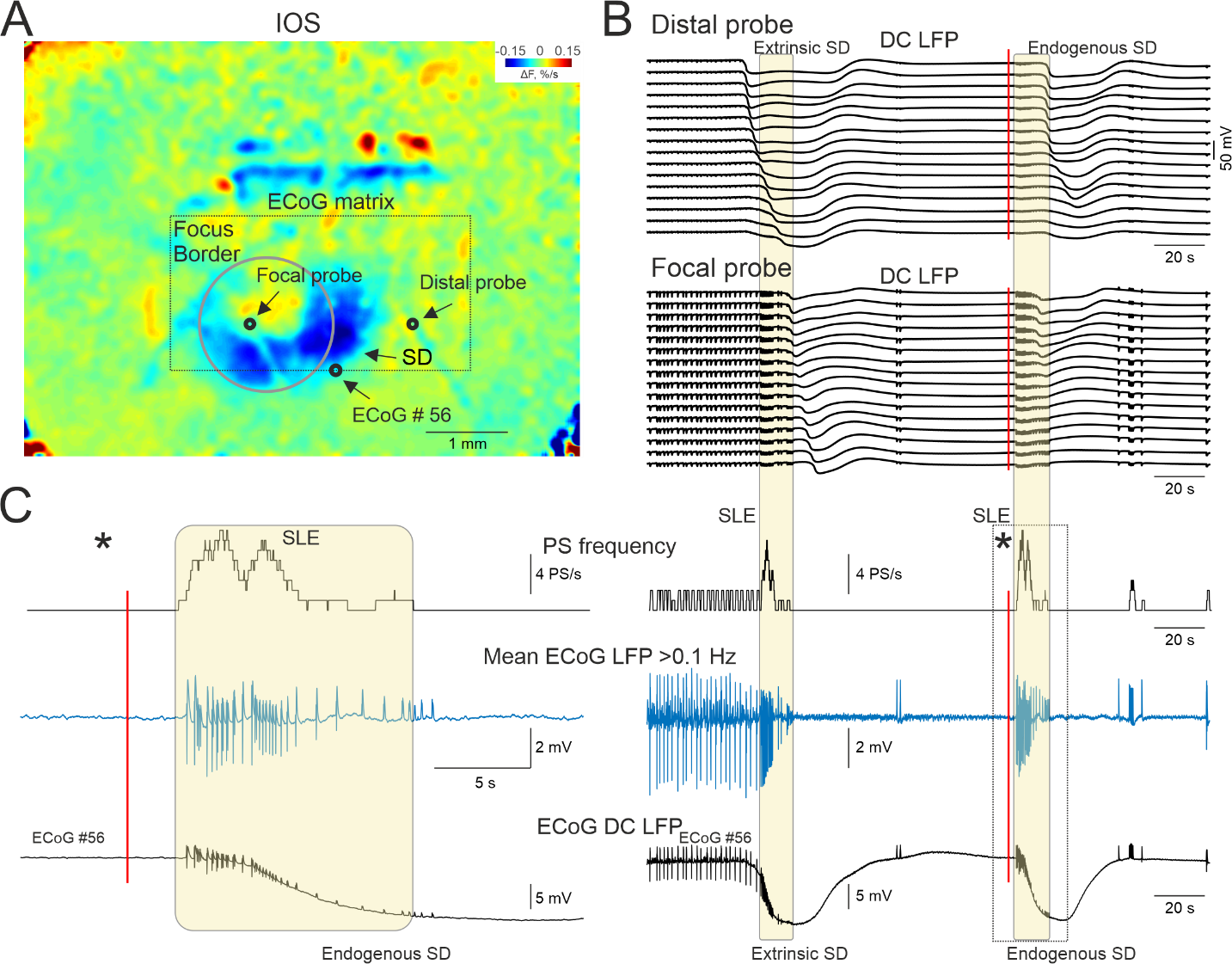


**Supplementary Figure S4. The case of endogenous SD that starts in the epileptic focus (related to the Supplementary Video S4).**

**(A)** A snapshot of IOS recordings at the onset of endogenous SD originating in the epileptic focus. The SD front (in blue) is indicated by the arrow. The epileptic focus border (concentric grey line) delineates the region with > half-maximal PS amplitude, and locations of the intracortical focal and distal silicon probes and of one of the ECoG electrodes (#56) are indicated by the circles. **(B)** Top, DC-LFPs recorded at different cortical depth with the distal and focal silicon probes during two consecutive SDs: the first, extrinsic SD originated from the KCl application site and propagated from right to left; the second, endogenous SD originated within the epileptic focus and spread concentrically outwards. Below, corresponding PS frequency plot, the mean AC-ECoG LFP (highpass filtered at > 0.1Hz) and DC-ECoG LFP at ECoG#56. The vertical red line corresponds to the time point of the still frame in panel A. Note that both extrinsic and endogenous SDs are associated with SLE (yellow boxes). (**C**) SLE and DC-ECoG#56 LFP associated with endogenous SD (outlined by a dashed line box and an asterisk on panel B) on an expanded time scale. To see the full view, see the Supplementary Video S4.
